# Genetic architecture of prostate cancer in the Ashkenazi Jewish population

**DOI:** 10.1038/bjc.2011.307

**Published:** 2011-08-09

**Authors:** J Vijai, T Kirchhoff, D Gallagher, N Hamel, S Guha, A Darvasi, T Lencz, W D Foulkes, K Offit, R J Klein

**Affiliations:** 1 Clinical Genetics Service, Department of Medicine, Memorial Sloan-Kettering Cancer Center, New York, NY 10065, USA; 2 Program in Cancer Genetics, Departments of Oncology and Human Genetics, McGill University; and Research Institute of the McGill University Health Center, Montreal, Quebec H2W 1S6, Canada; 3 Department of Psychiatry, Division of Research, The Zucker Hillside Hospital Division of the North Shore – Long Island Jewish Health System, Glen Oaks, NY 11004, USA; 4 The Institute of Life Sciences, The Hebrew University of Jerusalem, Jerusalem 91904, Israel; 5 Program in Cancer Biology and Genetics, Memorial Sloan-Kettering Cancer Center, 1275 York Avenue, Box 337, New York, NY 10065, USA

**Keywords:** SNPs, prostate cancer, association studies

## Abstract

**Background:**

Recently, numerous prostate cancer risk loci have been identified, some of which show association in specific populations. No study has yet investigated whether these single nucleotide polymorphisms (SNPs) are associated with prostate cancer in the Ashkenazi Jewish (AJ) population.

**Methods:**

A total of 29 known prostate cancer risk SNPs were genotyped in 963 prostate cancer cases and 613 controls of AJ ancestry. These data were combined with data from 1241 additional Ashkenazi controls and tested for association with prostate cancer. Correction for multiple testing was performed using the false discovery rate procedure.

**Results:**

Ten of twenty-three SNPs that passed quality control procedures were associated with prostate cancer risk at a false discovery rate of 5%. Of these, nine were originally discovered in studies of individuals of European ancestry. Based on power calculations, the number of significant associations observed is not surprising.

**Conclusion:**

We see no convincing evidence that the genetic architecture of prostate cancer in the AJ population is substantively different from that observed in other populations of European ancestry.

Recent studies have identified numerous single nucleotide polymorphisms (SNPs) that modify an individual's risk of developing prostate cancer (Amundadottir *et al*, 2006; Eeles *et al*, 2008; Gudmundsson *et al*, 2007a; Gudmundsson *et al*, 2008; Gudmundsson *et al*, 2007b; Haiman *et al*, 2007; Robbins *et al*, 2007; Thomas *et al*, 2008). Although some investigators have considered the possibility of heterogeneity between ethnic groups, where a SNP shows a different effect on prostate cancer risk depending on the population being studied, these studies only considered ethnic groups with different continents of ancestral origins (Haiman *et al*, 2007; Waters *et al*, 2009; Yamada *et al*, 2009; Hooker *et al*, 2010; Zheng *et al*, 2010). As alleles of numerous SNPs are known to vary in frequency across Europe (Bersaglieri *et al*, 2004), and population substructure is consistently observed in Americans with ancestry from different locations in Europe (Price *et al*, 2008; Tian *et al*, 2008), there is a possibility that prostate cancer risk alleles may have different effects in different populations of European ancestry.

Ashkenazi Jews are Jews whose ancestors come primarily from central and eastern Europe; the majority of North American Jews and a large proportion of Israeli Jews are of Ashkenazi ancestry. The global linkage disequilibrium (LD) profiles of the Ashkenazi Jewish (AJ) population do not seem to differ significantly from that of other populations of European ancestry. However, it has been suggested that there may be significant local differences in allele frequencies and haplotype structure between the Ashkenazi population and other European populations, including at loci associated with common cancer (Gold *et al*, 2008; Olshen *et al*, 2008; Price *et al*, 2008; Tian *et al*, 2008). Therefore, examination of known prostate cancer risk SNPs in the AJ population provides a unique opportunity to test for genetic heterogeneity at these loci among individuals of European ancestry.

Here, we report the results of a case–control association study in the AJ population of 29 previously identified prostate cancer SNPs. Our data argue against the hypothesis that risk alleles for prostate cancer generally have different effects in the Ashkenazi and non-Ashkenazi European ancestry populations.

## Materials and methods

Case and control DNA samples were obtained under IRB-approved protocols. Specifically for the samples from the Israeli blood bank, corresponding institutional review boards and the National Genetic Committee of the Israeli Ministry of Health approved the studies. The DNA samples from 963 prostate cancer cases were used in this study. Of these, 885 cases presented at Memorial Sloan-Kettering Cancer Center (MSKCC) with histologically confirmed prostate cancer and report all four grandparents were from Eastern Europe and Jewish. An additional 78 cases from Montreal, a Canadian metropolitan area, for whom both parents were of Ashkenazi ancestry were included. These patients were treated for prostate cancer at one of the three McGill University affiliated hospitals: the Royal Victoria and Montreal General Hospital sites of the McGill University Health Center and the Jewish General Hospital. Control DNA was collected from 1854 healthy men in New York and Israel – 419 participants in New York Cancer Project (Mitchell *et al*, 2004), 194 samples from the National Laboratory for the Genetics of Israeli Populations (NLGIP) (www.tau.ac.il/medicine/NLGIP/nlgip.htm), and 1241 healthy individuals from the Israeli Blood Bank. All controls self-report that all four grandparents are of Ashkenazi ancestry. These samples have been previously described (Kirchhoff *et al*, 2004; Shifman *et al*, 2008; Tischkowitz *et al*, 2008). The cases range in age from 26 to 94 (mean 68, s.d., 8.3). The controls range in age from 18 to 98 (mean 46, s.d., 15.2).

A total of 29 SNPs of interest were identified based on previous reports of association with prostate cancer risk from recent genome-wide association studies (GWAS) and other studies (Table 1). Most of these SNPs were selected on the basis of being reported in one of the GWAS papers as being significantly associated with prostate cancer risk. We also included numerous SNPs at 8q24 that were discovered in follow-up studies of this locus after its initial identification by linkage and association. Finally, we also included a SNP (rs7008482) reported as a prostate cancer risk SNP in the African-American population to see if this SNP had an effect in the Ashkenazi population despite not being associated in other European populations studied.

Samples from MSKCC, McGill University, the NY Cancer Project, and the NLGIP were genotyped using the Sequenom MassArray technology at MSKCC. This includes all cases and 31% of the controls. We designed two multiplex assays to genotype all 29 SNPs using Assay Design software (Sequenom, San Diego, CA, USA). PCR amplification and extension were performed using Sequenom iPLEX Gold reagents as per the manufacturer's protocol and analysed on the Sequenom MassARRAY system (Sequenom). Genotypes were called using the Typer 4.0 software package (Sequenom).

For quality control on the Sequenom data, we first manually inspected the cluster plots. Then, the data was processed with PLINK (Purcell *et al*, 2007). In all, 112 individuals with more than 20% missing data were removed. All SNPs had <20% missing data and showed no significant deviation from Hardy–Weinberg equilibrium in controls (*P*>0.01; Table 2). Six SNPs had significant differences in genotype calling rate between cases and controls (*P*<0.01; FDR<0.05) and were therefore removed from further consideration.

The controls from the Israeli blood bank were processed separately as they were initially genotyped genome wide as part of a separate study not related to cancer. These samples were fully anonymised immediately after collection and subsequently genomic DNA was extracted from blood samples by using the Nucleon kit (GE Healthcare, Piscataway, NJ, USA). The samples were genotyped on the Illumina HumanOmni1-Quad arrays (Illumina, San Diego, CA, USA) according to manufacturer's specifications under protocols approved by the Institutional Review Board of the North Shore-LIJ Health System. SNPs were filtered on the following bases: call rate <98%, minor allele frequency <0.02 and Hardy–Weinberg exact test *P*<0.000001. The samples were filtered based on cryptic identity and first- or second-degree relatedness using pairwise identity-by-decent (IBD) estimation (PI_HAT >0.20) in PLINK with 128 403 LD pruned (*r*^2^>0.2) genome-wide SNPs and population stratification using Principal Component Analysis with Ancestry Informative Markers specific for the AJ population.

Of the 23 SNPs that passed quality control from the Sequenom genotyping, 20 were directly genotyped on the Illumina chip. The remaining three SNPs were analysed only with the data from the Sequenom genotyping. Association analysis was performed in PLINK using logistic regression. Regression was performed twice, once without an adjustment for age and once with an adjustment for age of either diagnosis (cases) or sample collection (controls). Multiple testing was accounted for by holding the false discovery rate to be 5% using the Benjamini–Hochberg procedure (Benjamini and Hochberg, 1995).

To compute the power to detect association for each SNP, we assumed the previously reported odds ratio (OR), allele frequencies in our control population, and a sample size based on the number of successfully genotyped cases and controls. We used a previously reported method to compute the power at a significance level of 0.05 (Klein, 2007).

As a reference population of non-Ashkenazi white Americans, we used the GWAS data from the CGEMS Prostate Cancer GWAS – Stage 1 – PLCO (phs000207.v1.p1) in dbGaP (http://www.ncbi.nlm.nih.gov/gap), removing duplicate individuals. To test for the heterogeneity of the OR between the CGEMS data and our data, we used the Breslow–Day test as implemented in PLINK.

## Results

In the current study, we genotyped 29 SNPs previously reported as being associated with prostate cancer risk in 963 AJ prostate cancer cases and 613 AJ controls. The overall genotype call rate (fraction of genotypes for which a call is made) was 95%. After quality control (QC) filtering the Sequenom data as described in the methods, resulting in 23 SNPs that pass QC, we added data from 20 SNPs in 1241 male AJ controls genotyped with the Illumina Omni-1 Quad platform. As some controls came from Israel and some from the United States, we first queried if we observed allele frequency differences between AJ controls based on origin. Although two SNPs showed nominal differences in allele frequencies (rs9364554 and rs4242384; *P*<0.05), neither of these differences were significant after correcting for multiple testing. We were also concerned that the use of different genotyping platforms could lead to errors in our results. To test for this, we compared allele frequencies between individuals genotyped on the Illumina and Sequenom platforms and observed no differences (all nominal *P*>0.3).

We tested for association in 875 cases and 1810 controls total under an additive model. Before adjusting for age, 12 SNPs were nominally associated with prostate cancer risk (*P*<0.05; Table 3). Of these, 10 were significant at a false discovery rate of 5%. Among the 12 significant SNPs, only one – rs7008482 – shows a direction of effect opposite from that which was previously reported. As the SNP was identified in a case–control study of African-American men, we queried what effect this SNP had in the stage 1 data from the CGEMS prostate cancer GWAS of white Americans (Yeager *et al*, 2007). Although not significantly associated with risk (*P*=0.2), this SNP has the same direction of effect that we observe in the Ashkenazi population (OR=0.92; 95% CI=0.81–1.04). We next queried whether adjusting for age would influence these results. After removing 26 individuals without age information and adjusting for age as a covariate, nine SNPs were nominally significant (*P*<0.05), of which three are significant at a false discovery rate of 5% (Table 3). Notably, seven SNPs are nominally significant both with and without age adjustment.

We next wished to query if we could observe any heterogeneity between the effect size we observed in the Ashkenazi population and the effects observed in other populations of European ancestry. To do so in a systemised way, we used the stage 1 CGEMS data. There are 18 SNPs that we tested here that are also present in the CGEMS data. Of these, only one (rs4962416) shows heterogeneity (*P*=0.002). Although this SNP is associated with prostate cancer risk in the CGEMS stage 1 study (OR=1.3; 95% CI=1.2–1.5), we observe no evidence for association in the AJ population (OR=1.0; 95% CI=0.9–1.1).

For several of the SNPs, we did not replicate the association with prostate cancer risk observed in a number of prior studies (Kim *et al*, 2010). We note that the minor allele frequencies (MAF) at most of these SNPs in controls differ by at least 5 percentage points between the original report and our AJ individuals, and for seven SNPs the MAFs differ by at least 10 percentage points. As these differences in MAF could influence our power to replicate the previous findings, we computed the power using initially observed ORs from prior studies for each SNP along with the MAF observed in the AJ controls. We found that out of the nine SNPs for which we do not observe association with prostate cancer risk, our power to detect an association ranges from 37 to 99% there is >80% power to detect association at four of these SNPs (Table 3). We next queried if we would expect to find 12 or fewer significant associations by chance given the computed powers. In 10 000 simulations, given the computed power, we expect to find 12 or fewer significant associations 16% of the time.

## Discussion

Here, in the AJ population, we have replicated the association with prostate cancer risk for many of the prostate cancer risk SNPs tested. Overall, the effect of these SNPs in the AJ population is similar to that previously reported with the discovery of the SNPs. However, there are some SNPs for which we did not replicate the previously reported association despite having adequate power to do so. One potential explanation is the ‘winner's curse’, in which the first report of an association overestimates the magnitude of effect, leading to an inflated power estimation. In fact, for rs10486567, our estimate of 99% power was based on an OR of 0.74 as initially reported (Thomas *et al*, 2008). A more recent replication study suggests a more modest effect size of 0.84, which would result in only 80% power to detect the association in the present study (Prokunina-Olsson *et al*, 2010). Another possible explanation is that a previously reported risk SNP is truly not associated with prostate cancer in the AJ population, either due to differences in linkage disequilibrium patterns or the absence of the functional variant in the AJ population. This may explain the lack of association between rs16901979 and prostate cancer in the AJ population, as the observed OR for this rare SNP was 1.0 in our study.

Of the significant results, only one SNP – rs7008482 – showed a different direction of effect than that which has been previously reported. This SNP was first identified as a prostate cancer risk allele in the African-American population (Robbins *et al*, 2007). In a well-powered study of Japanese men, no evidence for association between this SNP and prostate cancer was observed (Yamada *et al*, 2009). Similarly, despite being present on the genome-wide genotyping chips used in several prostate cancer GWAS, this SNP was never reported as being associated with prostate cancer in European populations (Eeles *et al*, 2009; Gudmundsson *et al*, 2009; Yeager *et al*, 2009), though it does have the same direction of effect in the CGEMS stage 1 data (white American individuals) as we observe in the Ashkenazi population. As the direction of effect is opposite from that previously reported, more studies will be needed to determine if this is a real prostate cancer risk SNP, perhaps tagging different functional alleles in different populations, or represents a false positive finding. Fine mapping this locus in the AJ and African-American populations could help shed light on this question.

Of the 18 SNPs also present in the CGEMS Stage 1 data set, only one (rs4962416) showed a significant heterogeneity of the OR between the CGEMS study and our AJ study. This SNP does not appear to be associated with prostate cancer in the AJ population. Although other SNPs appear to have ORs near 1, the weak effect of these SNPs in the initial reports makes it difficult to determine if these SNPs are truly not associated in the AJ population, illustrating population heterogeneity, or that our sample size is simply not large enough. Larger studies in which we are well powered to replicate the known prostate cancer associations will be necessary to answer this question. Furthermore, our sample size is likely too small to distinguish more subtle differences in the ORs between the populations.

We used controls from both the United States and Israel. Although we have previously found that AJ individuals from these two countries cluster similarly using principal components analysis (Gold *et al*, 2008), we nevertheless tested each SNP for allele frequency differences between Israeli and United States controls. As we did not find any significant difference, we do not think this is a major source of potential error in our study.

These results provide evidence of some differences in the genetic architecture of prostate cancer between the AJ population and other populations of European ancestry. However, these few differences are not enough evidence to argue that there are substantive differences in genetic susceptibility to prostate cancer between these populations. Further study of the genetics of prostate cancer in this unique population will be needed to understand to what extent genetic risk to prostate cancer is similar to that in other European populations and to what extent it is different.

## Figures and Tables

**Table 1:** Known prostate cancer risk SNPs successfully tested in this study

**SNP**	**Chr.**	**Gene**	**Alleles (Maj/Min)**	**MAF**	**Prev. OR**	**Citation**
rs721048	2	*EHBP1*	G/A	0.19	1.15	Gudmundsson *et al* (2008)
rs2660753	3	*CHMP2B*; *POU1F1*	C/T	0.11	1.18	Eeles *et al* (2008)
rs9364554	6	*SLC22A3*; *SLC22A2*; *LPAL2*; *LPA*	C/T	0.29	1.17	Eeles *et al* (2008)
rs10486567	7	*JAZF1*	G/A	0.23	0.74	Thomas *et al* (2008)
rs6465657	7	*LMTK2*; *BHLHB8*	T/C	0.46	1.12	Eeles *et al* (2008)
rs7008482	8	—	T/G	0.83	1.8	Robbins *et al* (2007)
rs1016343	8	—	C/T	0.18	1.37	Eeles *et al* (2008)
rs13254738	8	—	A/C	0.34	1.11	Haiman *et al* (2007)
rs16901979	8	—	C/A	0.031	1.79	Gudmundsson *et al* (2007a)
rs6983267	8	—	G/T	0.50	0.8	Yeager *et al* (2007)
rs7000448	8	—	C/T	0.39	1.14	Haiman *et al* (2007)
rs4242382	8	—	G/A	0.12	1.41	Thomas *et al* (2008)
rs4242384	8	—	A/C	0.09	1.88	Eeles *et al* (2008)
rs7920517	10	*MSMB*	G/A	0.48	0.82	Eeles *et al* (2008)
rs10993994	10	*MSMB*	T/C	0.60	0.8	Eeles *et al* (2008)
rs4962416	10	*CTBP2*	T/C	0.27	1.2	Thomas *et al* (2008)
rs7931342	11	—	G/T	0.49	0.84	Eeles *et al* (2008)
rs10896449	11	—	G/A	0.48	0.78	Thomas *et al* (2008)
rs4430796	17	*TCF2*	G/A	0.49	1.24	Gudmundsson *et al* (2007b)
rs7501939	17	*TCF2*	C/T	0.42	0.83	Gudmundsson *et al* (2007b)
rs1859962	17	—	G/T	0.54	0.8	Gudmundsson *et al* (2007b)
rs2735839	19	*KLK2*; *KLK3*	G/A	0.15	0.83	Eeles *et al* (2008)
rs5945572	X	*NUDT11*; *NUDT10*	G/A	0.35	1.24	Gudmundsson *et al* (2008)

Abbreviations: alleles=major/minor alleles; chr.=chromosome; gene=nearby gene as reported in the cited literature; MAF=allele frequency in the controls of the cited paper for the minor allele as observed in the Ashkenazi Jewish population; prev. OR=previous odds ratio for the SNP as cited by the given paper; SNP=single nucleotide polymorphism.

When MAF is >0.5, it indicates that the minor allele in the Ashkenazi Jewish population is the major allele in the study that initially reported the SNP.

**Table 2:** Genotype counts and deviation from Hardy–Weinberg equilibrium stratified by disease status and source study

			**Genotype counts (Hardy–Weinberg *P*)**				
**SNP**	**Alleles (Maj/min)**	**MAF in controls**	**MSKCC controls**	**NLGIP controls**	**Israeli blood bank controls**	**MSKCC cases**	**Montreal cases**
rs721048	G/A	0.14	7/99/303 (1)	2/46/112 (0.37)	30/299/912 (0.36)	21/214/575 (0.79)	2/11/51 (0.19)
rs2660753	C/T	0.24	27/156/226 (1)	14/50/95 (0.084)	69/445/727 (0.94)	57/297/456 (0.35)	1/21/42 (0.67)
rs9364554	C/T	0.19	10/117/282 (0.72)	10/55/95 (0.66)	51/386/804 (0.59)	32/272/506 (0.59)	5/19/40 (0.28)
rs10486567	G/A	0.29	42/143/224 (0.013)	12/57/91 (0.53)	112/513/614 (0.73)	60/342/408 (0.34)	5/24/34 (0.75)
rs6465657	T/C	0.41	77/188/142 (0.31)	34/82/44 (0.75)	200/578/459 (0.44)	165/386/259 (0.35)	13/27/24 (0.31)
rs7008482	T/G	0.38	68/179/160 (0.14)	22/64/71 (0.22)	175/598/464 (0.47)	104/339/346 (0.16)	5/21/38 (0.49)
rs1016343	C/T	0.19	10/130/269 (0.25)	6/47/107 (0.79)	48/372/819 (0.46)	38/282/489 (0.84)	3/23/38 (1)
rs13254738	A/C	0.41	64/204/141 (0.54)	29/70/61 (0.25)	218/572/447 (0.14)	153/425/232 (0.10)	9/34/21 (0.60)
rs16901979	C/A	0.04	1/34/374 (0.55)	0/10/147 (1)	ND	1/62/748 (1)	0/8/55 (1)
rs6983267	G/T	0.50	96/207/104 (0.77)	36/82/42 (0.87)	318/615/305 (0.82)	168/391/249 (0.52)	12/27/25 (0.43)
rs7000448	C/T	0.48	90/203/116 (1)	39/85/36 (0.53)	279/606/341 (0.77)	234/380/191 (0.14)	16/26/21 (0.21)
rs4242382	G/A	0.065	1/62/345 (0.49)	0/14/146 (1)	4/150/1087 (0.81)	8/125/677 (0.38)	0/5/59 (1)
rs4242384	A/C	0.062	1/57/351 (0.71)	0/12/148 (1)	ND	5/118/687 (1)	0/5/59 (1)
rs7920517	G/A	0.45	81/194/132 (0.55)	42/75/41 (0.53)	250/605/386 (0.65)	150/392/268 (0.77)	12/33/19 (0.80)
rs10993994	T/C	0.50	97/202/109 (0.84)	46/77/35 (0.87)	305/634/301 (0.46)	184/396/229 (0.62)	13/34/17 (0.80)
rs4962416	T/C	0.33	46/193/170 (0.51)	18/78/64 (0.49)	133/516/591 (0.21)	95/344/370 (0.27)	6/25/33 (0.76)
rs7931342	G/T	0.33	46/173/185 (0.57)	20/71/69 (0.86)	149/533/556 (0.23)	69/319/422 (0.44)	6/29/29 (1)
rs10896449	G/A	0.34	52/175/181 (0.38)	20/72/68 (0.86)	155/542/543 (0.28)	76/321/411 (0.27)	6/29/29 (1)
rs4430796	G/A	0.43	69/206/134 (0.54)	35/76/49 (0.63)	225/607/392 (0.73)	183/397/230 (0.67)	11/35/18 (0.46)
rs7501939	C/T	0.44	82/206/121 (0.77)	29/77/52 (1)	222/614/390 (0.49)	131/395/283 (0.77)	13/31/19 (1)
rs1859962	G/T	0.47	99/189/121 (0.14)	27/89/41 (0.11)	ND	157/392/262 (0.67)	14/36/14 (0.45)
rs2735839	G/A	0.18	8/119/281 (0.37)	8/42/110 (0.18)	50/372/818 (0.36)	30/210/570 (0.058)	3/22/39 (1)
rs5945572	G/A	0.25	97/5/307 (NA)	47/2/110 (NA)	303/0/936 (NA)	244/2/563 (NA)	17/0/47 (NA)

Abbreviations: MAF=minor allele frequency; min=minor; maj=major; MSKCC**=**Memorial Sloan-Kettering Cancer Center; NA=not applicable (X-chromosome SNP and all male study); ND=not done (no data is available on the Illumina chip for these individuals); NLGIP=National Laboratory for the Genetics of Israeli Populations; SNP=single nucleotide polymorphism.

Genotype counts are given as minor homozygote/heterozygote/major homozygote.

**Table 3:** Association of known prostate cancer risk SNPs in the Ashkenazi Jewish population

				**Unadjusted**		**Age adjusted**		
**SNP**	**Chr.**	**Gene**	**Alleles (Maj/Min)**	**OR (95% CI)**	***P***	**OR (95% CI)**	***P***	**Power**
rs721048	2	*EHBP1*	G/A	1.09 (0.93–1.27)	0.3	1.16 (0.94–1.44)	0.17	0.37
rs2660753	3	*CHMP2B*; *POU1F1*	C/T	1.04 (0.91–1.19)	0.55	0.98 (0.82–1.16)	0.78	0.65
rs9364554	6	*SLC22A3*; *SLC22A2*; *LPAL2*; *LPA*	C/T	1.1 (0.96–1.27)	0.19	1.01 (0.83–1.22)	0.94	0.54
rs10486567	7	*JAZF1*	G/A	0.98 (0.86–1.11)	0.71	1.14 (0.97–1.35)	0.12	1
**rs6465657**	7	*LMTK2*; *BHLHB8*	T/C	1.14 (1.01–1.27)	0.026	1.12 (0.96–1.3)	0.15	0.47
**rs7008482**	8	—	T/G	0.84 (0.74–0.94)	0.0036	0.87 (0.74–1.02)	0.08	1
**rs1016343**	8	—	C/T	1.24 (1.07–1.42)	0.0031	1.19 (0.98–1.43)	0.075	0.98
**rs13254738**	8	—	A/C	1.19 (1.06–1.33)	0.0039	1.15 (0.98–1.34)	0.086	0.42
rs16901979	8	—	C/A	1.01 (0.69–1.48)	0.94	0.94 (0.58–1.53)	0.8	0.77
**rs6983267**	8	—	G/T	0.81 (0.72–0.91)	0.00026	0.83 (0.72–0.97)	0.018	0.97
**rs7000448**	8	—	C/T	1.2 (1.07–1.34)	0.0021	1.18 (1.01–1.37)	0.033	0.61
**rs4242382**	8	—	G/A	1.31 (1.05–1.62)	0.015	1.63 (1.21–2.21)	0.0013	0.75
**rs4242384**	8	—	A/C	1.24 (0.92–1.68)	0.16	1.61 (1.09–2.36)	0.017	0.95
**rs7920517**	10	*MSMB*	G/A	0.92 (0.82–1.03)	0.17	0.84 (0.72–0.98)	0.026	0.92
**rs10993994**	10	*MSMB*	T/C	0.89 (0.79–1)	0.047	0.84 (0.72–0.98)	0.022	0.97
rs4962416	10	*CTBP2*	T/C	1 (0.89–1.13)	0.96	0.95 (0.81–1.11)	0.51	0.82
**rs7931342**	11	—	G/T	0.8 (0.7–0.9)	0.0003	0.79 (0.67–0.92)	0.0035	0.82
**rs10896449**	11	—	G/A	0.8 (0.71–0.91)	0.00044	0.8 (0.68–0.93)	0.0054	0.99
**rs4430796**	17	*TCF2*	G/A	1.17 (1.04–1.31)	0.0092	1.19 (1.02–1.39)	0.023	0.95
rs7501939	17	*TCF2*	C/T	0.9 (0.8–1.01)	0.065	0.95 (0.81–1.11)	0.53	0.89
rs1859962	17	—	G/T	0.89 (0.77–1.04)	0.14	0.92 (0.76–1.11)	0.4	0.83
rs2735839	19	*KLK2*; *KLK3*	G/A	0.91 (0.79–1.06)	0.24	0.89 (0.73–1.08)	0.24	0.73
**rs5945572**	X	*NUDT11*; *NUDT10*	G/A	1.3 (1.08–1.55)	0.0049	1.17 (0.92–1.48)	0.21	0.87

Abbreviations: alleles=major/minor alleles; Chr.=chromosome; gene=nearby gene as reported in the cited literature; min=minor; maj=major; OR=odds ratio for the minor allele; SNP=single nucleotide polymorphism; 95% CI=95% confidence interval.

SNPs in bold are significant at a nominal *P*<0.05 either without age adjustment, with age adjustment or both. Underlined *P*-values are significant at a false discovery rate of 5%.
